# Saving Mothers, Giving Life Approach for Strengthening Health Systems to Reduce Maternal and Newborn Deaths in 7 Scale-up Districts in Northern Uganda

**DOI:** 10.9745/GHSP-D-18-00263

**Published:** 2019-03-11

**Authors:** Simon Sensalire, Paul Isabirye, Esther Karamagi, John Byabagambi, Mirwais Rahimzai, Jacqueline Calnan

**Affiliations:** a University Research Co., LLC, Kampala, Uganda.; b U.S. Agency for International Development, Kampala, Uganda.

## Abstract

**Background:**

Uganda's maternal and newborn mortality remains high at 336 maternal deaths per 100,000 live births and 27 newborn deaths per 1,000 live births. The Saving Mothers, Giving Life (SMGL) initiative launched in 2012 by the U.S. government and partners, with funding from the U.S. President's Emergency Plan for AIDS Relief, focused on reducing maternal and newborn deaths in Uganda and Zambia by addressing the 3 major delays associated with maternal and newborn deaths. In Uganda, SMGL was implemented in 2 phases. Phase 1 was a proof-of-concept demonstration in 4 districts of Western Uganda (2012 to 2014). Phase 2 involved scaling up best practices from Phase 1 to new sites in Northern Uganda (2014 to 2017).

**Program Description:**

The SMGL project used a systems-strengthening approach with quality improvement (QI) methods applied in targeted facilities with high client volume and high maternal and perinatal deaths. A QI team was formed in each facility to address the building blocks of the World Health Organization's health systems framework. A community component was integrated within the facility-level QI work to create demand for services. Above-site health systems functions were strengthened through engagement with district management teams.

**Results:**

The institutional maternal mortality ratio in the intervention facilities decreased by 20%, from 138 to 109 maternal deaths per 100,000 live births between December 2014 and December 2016. The institutional neonatal mortality rate was reduced by 30%, while the fresh stillbirth rate declined by 47% and the perinatal mortality rate by 26%. During this period, over 90% of pregnant women were screened for hypertension and 70% for syphilis during antenatal care services. All women received a uterotonic drug to prevent postpartum hemorrhage during delivery, and about 90% of the women were monitored using a partograph during labor.

**Conclusions:**

Identifying barriers at each step of delivering care and strengthening health systems functions using QI teams increase partcipation, resulting in improved care for mothers and newborns.

## Abstract (precis)

Saving Mothers, Giving Life (SMGL) strengthened the health system in 7 districts in Northern Uganda through a quality improvement approach. Quality improvement teams removed barriers to delivering maternal and newborn health services and improved emergency care, reducing preventable maternal and newborn deaths in a post-conflict, low-resource setting.

## INTRODUCTION

Maternal and newborn mortality in Uganda remains unacceptably high.^1^ The maternal mortality ratio (MMR) is recorded at 336 maternal deaths per 100,000 live births.^2^ With funding from the U.S. President's Emergency Plan for AIDS Relief (PEPFAR) and the United States Agency for International Development (USAID), the Saving Mothers, Giving Life (SMGL) initiative was launched in 2012 with the Ministry of Health (MOH) in Uganda and Zambia to reduce such deaths. Most maternal and newborn deaths occur during labor, delivery, and the immediate postpartum period.^3^ Hence, strategies to address the deaths centered on the 3 major delays in accessing and using health care during these periods, namely, delays in seeking appropriate care, inability to access the most appropriate care in a timely manner, and inconsistencies in the quality of care provided at health facilities.

**FIGURE 1:**
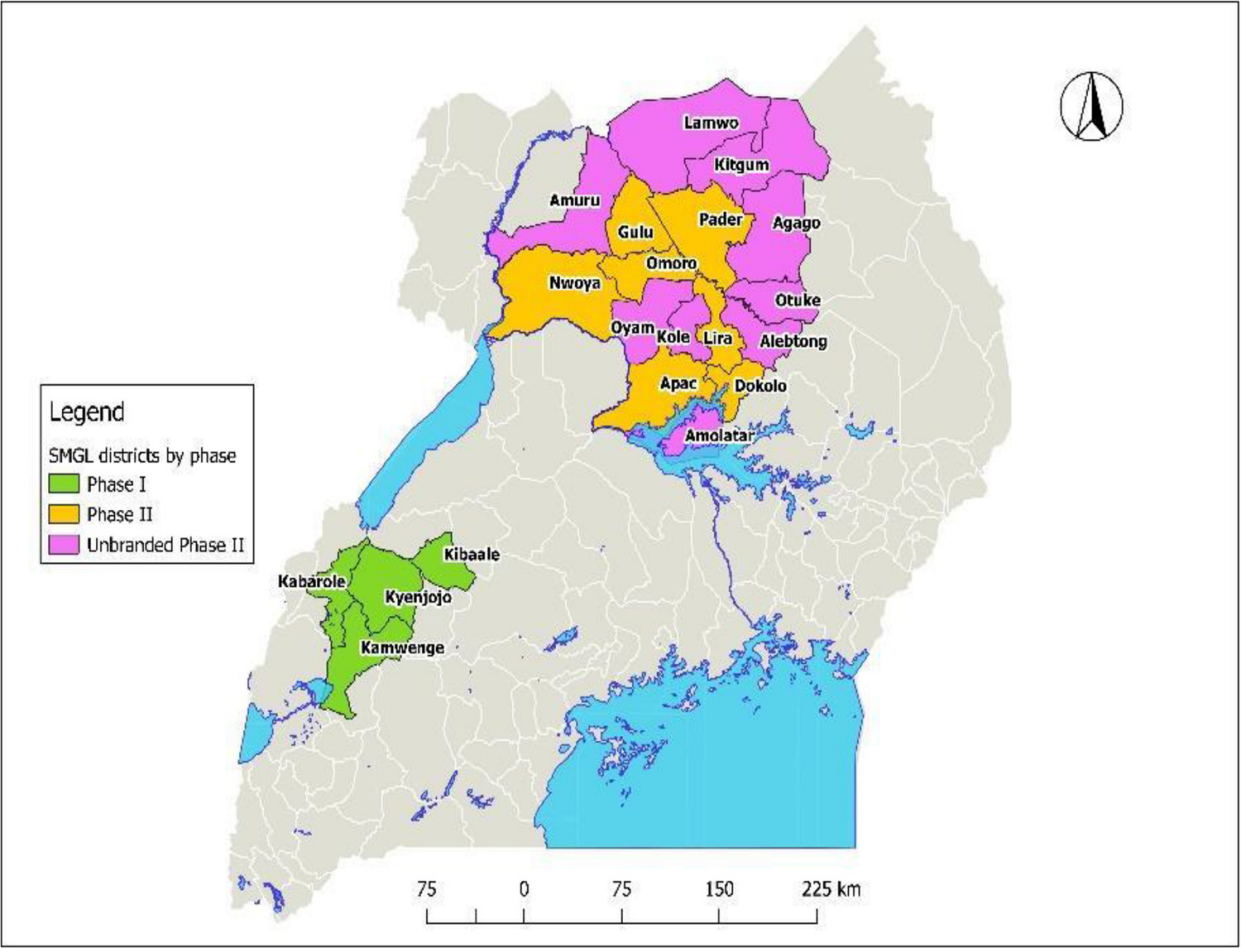
Map of Uganda Showing the Various Phases of SMGL Implementation Abbreviation: SMGL, Saving Mothers, Giving Life.

The SMGL initiative was implemented in 2 phases. Phase 1 was the proof of concept, implemented in 4 districts of Western Uganda—Kyenjojo, Kamwenge, Kabarole, and Kibaale—between June 2012 and December 2013. The population of the 4 districts was estimated to be 1.3 million in 2013.^2^ Phase 2 continued efforts in the 4 Phase 1 districts and scaled up the best practices developed in Phase 1 to 7 more districts in Northern Uganda—Nwoya, Gulu, Omoro (recently carved out of Gulu), Pader, Lira, Dokolo, and Apac—between February 2015 and December 2016. The 7 new districts had an estimated population of 1,812,800 between January 2016 and September 2017. The learnings from the SMGL-supported districts in Northern Uganda were further spread to 9 surrounding districts in the same region (Oyam, Alebtong, Amolatar, Kitgum, Agago, Amur, Lamwo, Kole, and Otuke) starting in 2016, supported under the maternal and child health PEPFAR platform (Figure 1). These districts had an estimated population of 1,773,600 in 2016.

**FIGURE 2:**
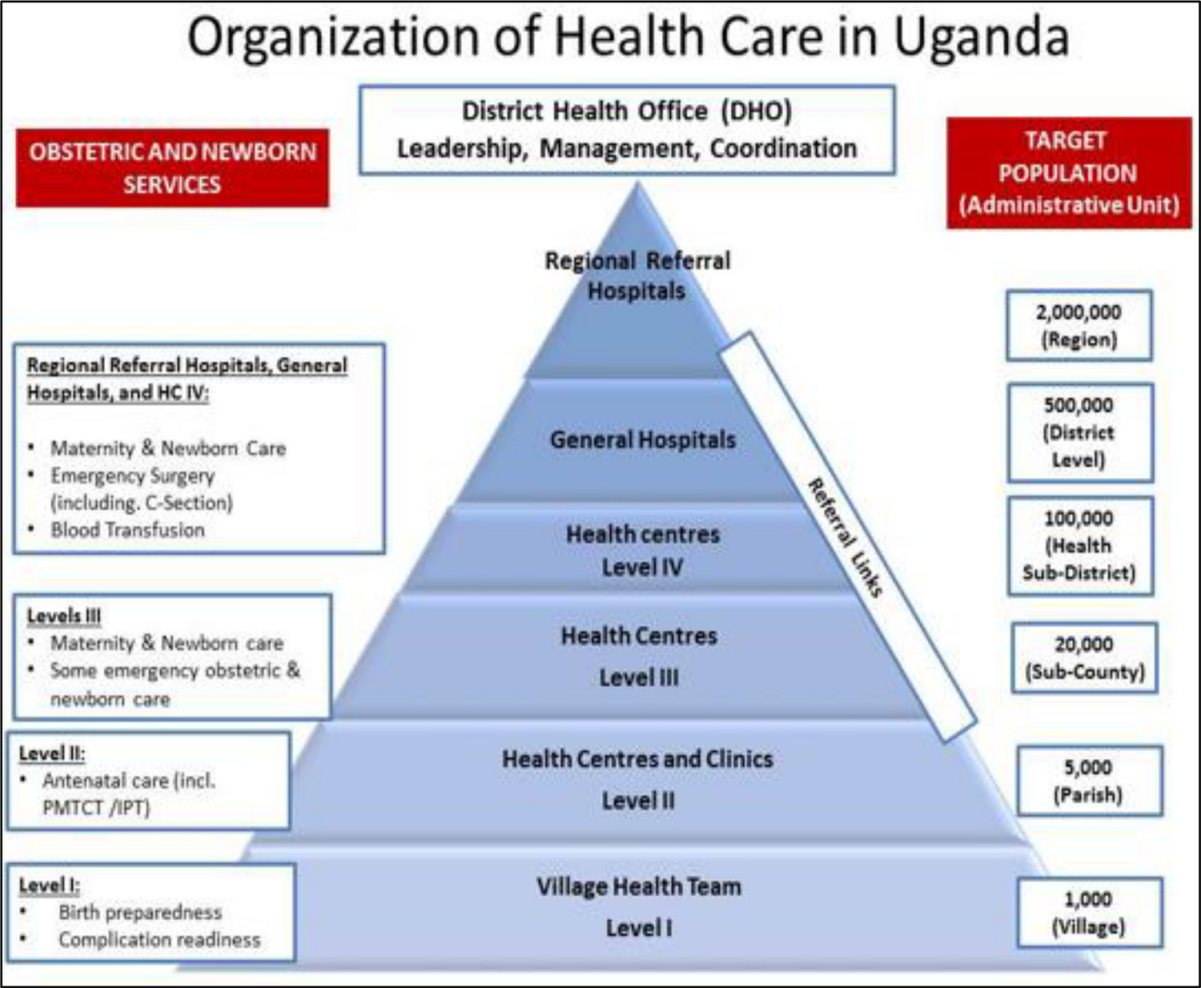
Levels of Health Care Service Delivery in Uganda Source: Northern Uganda Health Integration to Enhance Services (NU-HITES) Assessment Report for Emergency Obstetric Care in Northern Uganda, 2014. Abbreviations: C-section, cesarean section; HC, health center; IPT, intermittent preventive treatment; PMTCT, prevention of mother-to-child transmission (of HIV).

The scale-up phase in Northern Uganda was carried out in 3 waves. Wave I targeted 20 high-volume public and private not-for-profit facilities with more than 100 deliveries per month—where 64% of deliveries, 74% of newborn deaths, and 95% of maternal deaths occurred in 2013—and 144 surrounding communities.^4^ Wave II involved 60 medium-volume facilities with 50 to less than 100 deliveries per month, which included primarily third-level health centers (HC IIIs) and an additional 370 communities within the catchment areas of the supported facilities. Wave III involved 38 low-volume facilities with less than 50 deliveries per month, including second-level health centers (HC IIs) that conduct deliveries (Figure 2). The wave-spread approach to implementation of quality improvement (QI) strategies was based on successes of QI in the early waves and availability of funding for implementing QI strategies within other intervention districts.

As in the initial scale-up, the implementation strategy for these new districts targeted all levels of the health system. The national level provided technical oversight and competency building. The district coordinated and supported application of technical knowledge, decision making, and resource mobilization. Health facilities provided quality maternal and newborn health (MNH) services and strengthened emergency care functions. The community level created demand for services including referral tracking.

This paper describes the scale-up of SMGL in Northern Uganda starting in 2015 and the results as of December 2016. The first 7 scale-up districts (Phase 2) are referred to as the SMGL-supported districts and the last 9 scale-up districts, implemented under the maternal and child health PEPFAR platform, are referred to as the unbranded SMGL-supported districts. The latter did not have rigorous monitoring and evaluation, as no funds were allocated or staff dedicated to those activities. Our findings may help inform program managers and health care providers in other low-resource settings on the use of quality improvement methods to strengthen the health system to reduce preventable maternal and newborn deaths.

## PROGRAM DESCRIPTION

SMGL is a multi-partner initiative designed to rapidly reduce deaths stemming from pregnancy and childbirth through a comprehensive set of evidence-based interventions in high-mortality, low-resource settings. The initiative established an ambitious target of a 50% decline in the MMR within 1 year to address the need for accelerated progress to meet the fifth Millennium Development Goal (MDG5) of a 75% reduction in MMR by 2015. SMGL draws upon the investment and expertise of public and private organizations and existing infrastructure, partnerships, and services, including U.S. government platforms, for combating HIV/AIDS and improving maternal and child health.^5^

The scale-up Phase 2 in Northern Uganda involved spreading lessons from experience gained in reducing maternal and newborn deaths in the Phase 1 SMGL-supported districts in Western Uganda (Table 1). The scale-up used a wave-sequence model of spread wherein learning from early intervention sites was spread to surrounding sites.^6^ The spread sites were selected based on certain criteria, such as client volume in terms of deliveries per month, readiness to change (defined as the ability to adopt best practices from the learning phase), and leadership, in terms of willingness of the facility leadership to embrace QI activities in their respective facilities. The sites meeting these criteria were then organized into waves. Spread was implemented through coaching visits to the wave sites, engaging both coaches who supported the learning phase and champions from the learning phase sites. Each wave received a minimum of 3 monthly coaching visits before another wave was launched. Within a 12-month period, all wave sites were implementing the changes from the change package, an evidence-based set of best practices crucial to the improvement of an identified care process, such as the use of partograph for monitoring the third stage of labor.

**TABLE 1.** SMGL Interventions Implemented During Phase 1 and Scaled Up During Phase 2 to Reduce the 3 Delays in Northern Uganda

SMGL Interventions (Phase I)	SMGL Intervention Scaled Up to Phase 2 of Northern Uganda	Nonbranded SMGL Scale-Up (in Northern Uganda)
Increase awareness and seeking care for safe delivery to reduce the first delay	√	√
Training of village health teams to encourage birth preparedness and increase demand for facility-based delivery care	√	√
Community outreach activities to counsel women, families, local leaders, and community organizations	√	√
Distribution of mama kits to incentivize facility-based births	X	X
Community mobilization messages (e.g., radio, billboards, and newspaper articles) and drama skits	√	√
Promotion of demand- and supply-side financial incentives to facilitate women seeking, accessing, and using quality care services (e.g., transport and delivery care vouchers, user-fee reductions, and conditional cash transfers)	Use of saving groups to save for birth expenses	Use of saving groups to save for birth expenses
Increase access to quality health care services to reduce the second delay	√	√
Upgrade a sufficient number of public and private facilities with appropriate geographical positioning to provide—24 hours a day/7 days a week—clean and safe basic delivery services	√	√
Ensure that a minimum of 5 EmONC facilities are providing the recommended lifesaving obstetric interventions 24 hours a day/7 days a week	√	√
Hire a sufficient number of skilled birth attendants to consistently provide quality, respectful basic delivery care, diagnosis, and stabilization of complications	X	X
Create a consultative, protocol-driven, quality-assured, and integrated communication/transportation referral system available 24 hours a day/7 days a week that ensures women with complications reach emergency services within 2 hours	√	√
Improve quality, appropriate, and respectful care to reduce the third delay	√	√
Train health professionals in emergency obstetric care, including obstetric surgeries	√	√
Ensure mentoring of newly hired personnel and supportive supervision	√	√
Strengthen supply chains for essential supplies and medicines	√	√
Ensure implementation of quality effective interventions to prevent and treat obstetric complications	√	√
Introduce sound managerial practices utilizing “short-loop” data feedback and response, to ensure reliable delivery of quality essential and emergency maternal and newborn care	√	√
Strengthen maternal mortality surveillance in communities and facilities, including timely, no-fault medical death reviews performed in follow-up to every institutional maternal death with cause of death information used for ongoing monitoring and quality improvement	√	√
Promote a government-owned health management information system that accurately records every birth, obstetric and newborn complication and treatment provided, and birth outcome at public and private facilities in the district	√	√

Abbreviations: EmONC, emergency obstetric and newborn care; SMGL, Saving Mothers, Giving Life.

√, SMGL interventions were implemented.

X, SMGL interventions were not implemented.

Coaches were selected according to defined criteria: they had been champions for improvement of work in maternal, newborn, and child health (MNCH) in their respective facilities, were located in the intervention areas, had expressed willingness to mentor others, and had been involved in health services for MNH. The coaches were recruited across the intervention districts to support facilities within a defined geographical area. They were linked to the technical support supervision structure and included gynecologists, pediatricians, medical officers, and midwives at all levels for continuity of the improvement activities (Figure 2).

**FIGURE 3:**
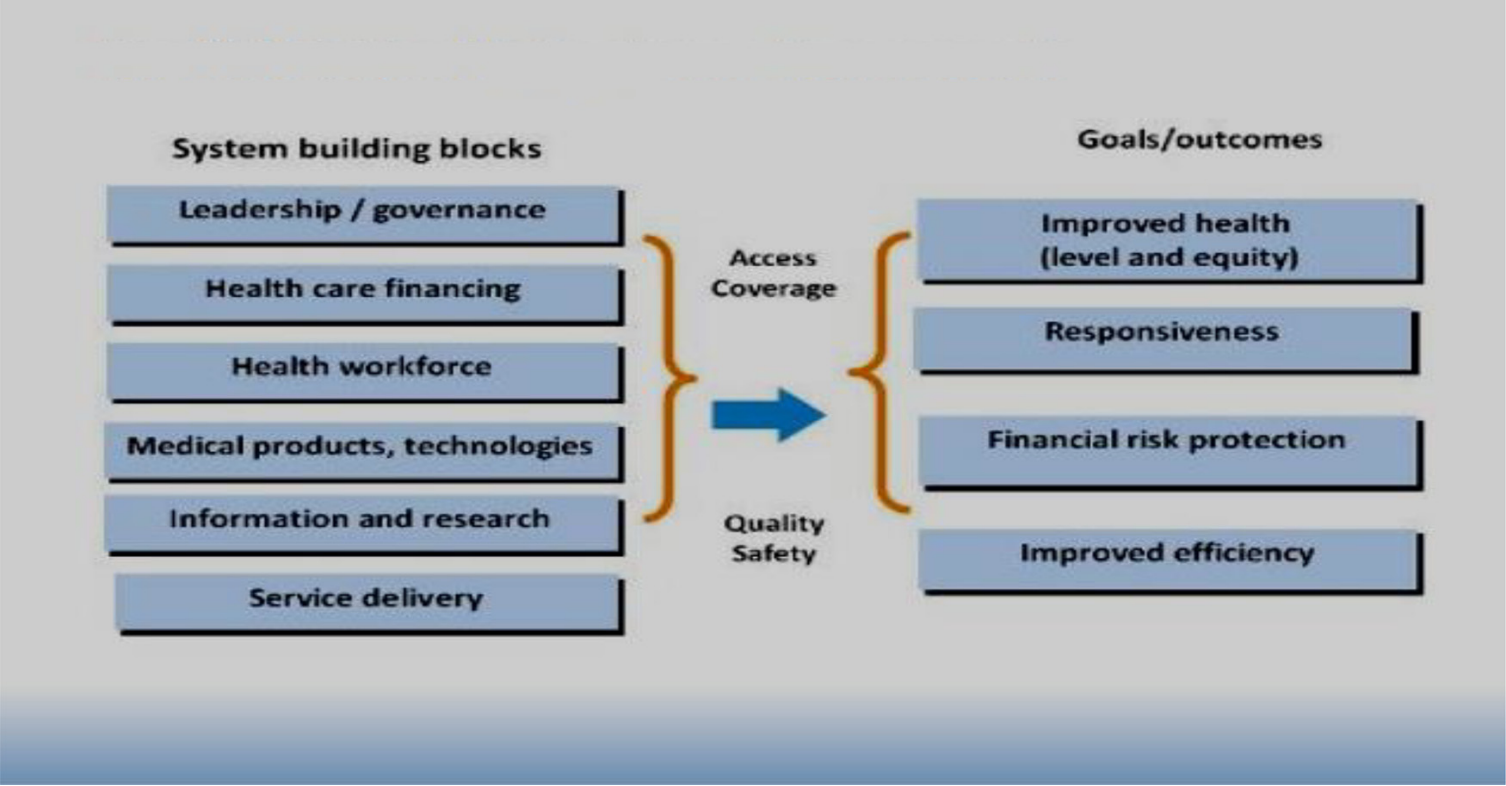
The WHO Health Systems Framework

With engagement of key MNH stakeholders at national, district, and facility levels in Northern Uganda, SMGL addressed gaps, impeding delivery of quality antenatal care (ANC), labor and delivery, and newborn care services, through formation of QI teams targeting frontline health care providers and managers involved in the provision of maternal and newborn care services, mainly medical officers, midwives, records officers, laboratory technicians, pharmacists/dispensers, and facility managers. These teams were situated in maternity units and met on a weekly or monthly basis to fulfill their improvement objectives to track weekly performance, share results with the facility administration, and share personal experiences in quarterly learning meetings. The interventions for strengthening the health system included regular skill building through coaching and mentorship visits, quarterly peer-to-peer learning meetings, maternal and perinatal death reviews to identify and avert similar causes of deaths, regular performance and data improvement meetings at facility and district levels, fourth-level health centers (HC IVs) updated to provide comprehensive emergency obstetric and newborn care (CEmONC), and establishment of skills labs at health facilities. The strategy for addressing each health system building block to improve MNH^7^ (Figure 3) is described next.

SMGL addressed gaps impeding delivery of quality ANC, labor and delivery, and newborn care services through the formation of QI teams.

### Leadership and Governance

The project engaged staff from the MOH Reproductive and Child Health and Health Promotion divisions to provide technical updates and implement maternal and perinatal death review (MPDR) tools in the Northern Ugandan project districts. MOH designated 12 maternal and newborn technical mentors from teaching and referral institutions in the region, who then dedicated 25% of their time every month to support program activities facilitated by the SMGL project. They participated in technical supervision of facilities, training of MPDR committees to conduct maternal and perinatal audits, and support for improvement teams. The mentors from national and regional levels supported skills development of health care providers at various levels of health facilities (HC IIIs, HC IVs, and hospitals). They were advised to include this activity as part of their work plans for supporting facilities without interrupting routine activities. With time, these roles would be integrated in the routine technical supervision of facilities by all levels for continuity. These mentors continued to work with the SMGL project staff over the year to improve demand, access, and quality care through monthly mentorships at health facility and community levels. The project facilitated the mentors' transportation to different facilities and communities within their geographical areas of operation.

**FIGURE 4:**
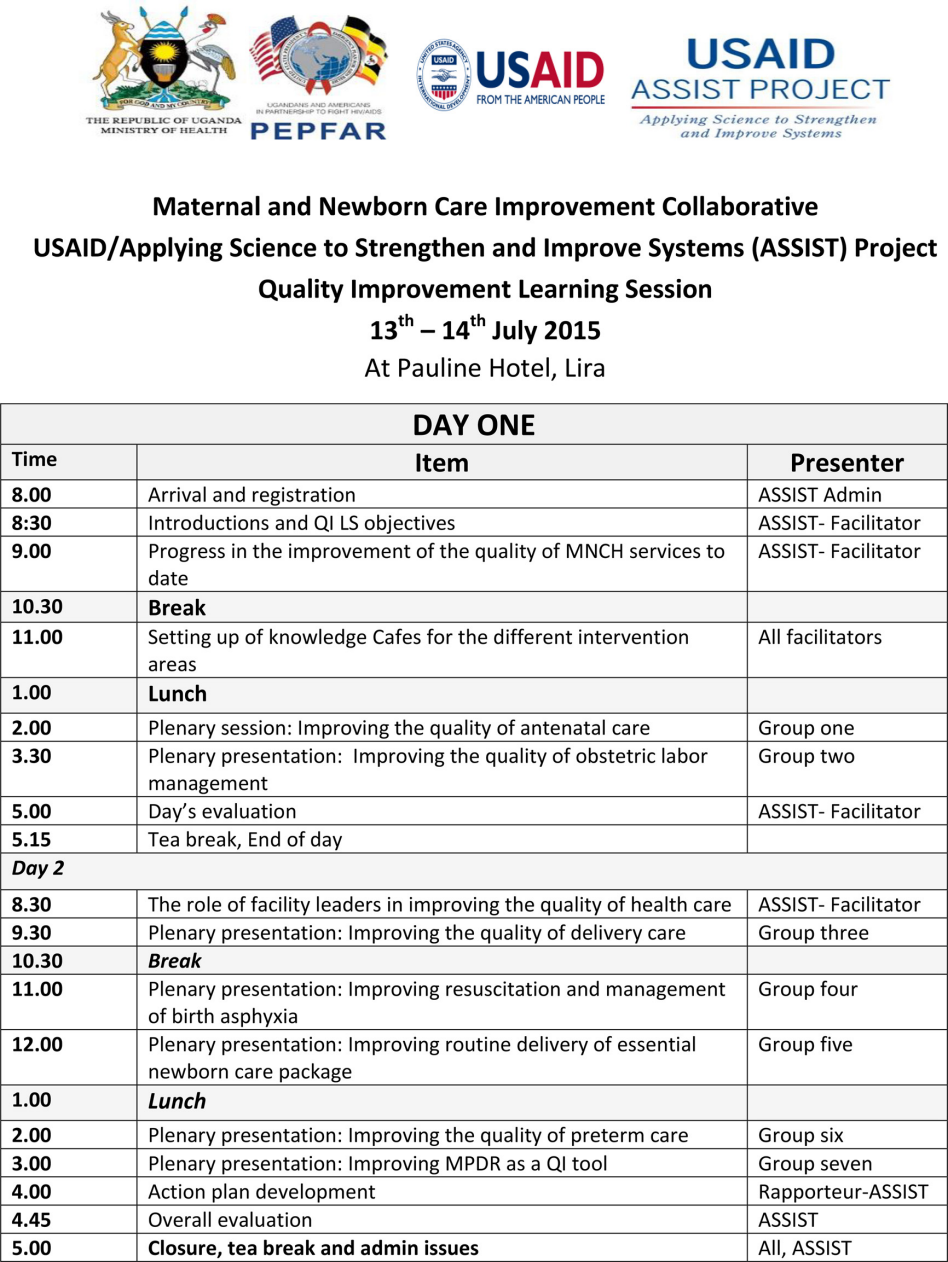
Agenda for a Learning Meeting Held for Phase II Facilities in Northern Uganda Involving Facility, District, and Implementing Partner Stakeholders Abbreviations: LS, learning session; MNCH, maternal, newborn, and child health; MPDR, maternal and perinatal death review; QI, quality improvement.

Within each intervention district in Northern Uganda, project staff worked under the district health officer to engage the district health team, partners, and political leaders in quarterly coordination and performance improvement meetings; make designated CEmONC facilities functional through rehabilitation and re-equipping of infrastructure; reallocate human resources to understaffed high-volume facilities; conduct monthly coaching/mentorship and supervisory visits; and hold quarterly district-level MPDR meetings. The quarterly meetings involved coordinating efforts aimed at reducing maternal and newborn mortality, reviewing data, and discussing performance improvement based on district-specific barriers to accessing quality MNCH services (Figure 4). Since QI activities for SMGL involved leadership across district, facility, and community levels, the improvements in uptake of services and reductions in mortality were used as an indirect estimate of the influence of leadership and governance.

### Health Care Financing

The respective district health officers, facility managers, and project staff mobilized resources from local NGOs and district councils to address the identified facility-level gaps. The district health officers with other district technical leaders quantified facility-specific gaps and presented proposed budgets to their district councils for allocation of funds to address those gaps. For example, funds were allocated to renovate and make functional the operating theaters of Lalogi and Awach (Gulu district), Ogur (Lira district), and Pajule (Pader district) HC IVs. In specific locales, they also mobilized local NGOs (e.g., Straight Talk organization in Gulu district), which provided solar lighting kits to 3 HC IIIs. In-charges of health facilities were mobilized to budget for photocopying and printing of partograph sheets and purchase basic equipment, such as blood pressure machines, using primary health care funds—monies allocated to each health facility per quarter from government. Delays in fund disbursement for primary health care were counteracted with resource mobilization from NGOs.

### Health Workforce

The Pader district health office received support to send a staff member for training in anesthesia and to reallocate midwives to lower-level facilities that did not have midwives (Box 1). In other facilities, facility staff, including medical officers and midwives, were organized into QI teams and coached monthly by a team of trained local coaches/mentors—champions/reproductive health trainers within the intervention districts—and project staff to identify and address critical gaps in care processes. A local support network of technical and improvement experts from Gulu and Lira regional referral hospitals and universities, plus district-based reproductive health trainers and champions from health facilities, was established to visit health facilities monthly to address knowledge and skills gaps and to motivate health care providers. This network was further integrated into the routine technical supervision for health workers at all health facilities. Onsite continuous medical education was institutionalized and used to build and improve provider skills and aid retention of skills over time.

BOX 1 **Health Workforce Development for Pader District**

What worked for Pajule Level IV Health Center?

1. Human resource management: Identified and trained an interested health worker in anesthesia
2. District support for in-service training: Lobbied for funds from IntraHealth International to support the training of a staff member and also granted him a paid leave
3. Retention of staff member: Promoted the new anesthetic officer in order to retain him

During 2015–2016, 303 of the total 450 relevant medical personnel were trained in emergency obstetric and newborn care, long-acting family planning methods, and newborn resuscitation. To enhance skills in newborn resuscitation, Helping Babies Breathe skills labs were established in 14 facilities at regional, general hospital, and HC IV levels. Monthly onsite technical mentorship and QI coaching sessions supplemented this capacity building, reaching 450 health workers in the 7 initial Phase 2 SMGL-supported districts every month at HC IIIs, HC IVs, and hospitals. Project and district management teams convened quarterly peer-to-peer learning sessions, bringing together representatives from the 118 facilities to share their results and challenges.

Two-thirds of relevant medical personnel were trained in emergency obstetric and newborn care, long-acting family planning methods, and newborn resuscitation/Helping Babies Breathe.

### Medical Products and Technologies

The staff responsible for supplies and drug management—pharmacists, dispensers, and storekeepers in supported facilities—were coached on the specific drugs and supplies to order and the ordering schedules of the national medical stores, as a means of preventing stock-outs. SMGL coordinated with the medicine management supervisors, 1 per health subdistrict, in each district to redistribute drugs and supplies to facilities with stock-outs. In addition, routine maintenance and repairs of medical equipment were facilitated for lower-level health facilities through coordination with the medical engineering departments at the 2 regional referral hospitals of Gulu and Lira. Following a review of equipment needed in participating facilities, sites in Northern Uganda received donated equipment for maternal and newborn care, including radiant warmers, bulb syringes, oxygen concentrators, emergency drug trollies, anesthetic machines, wheelchairs, delivery/postnatal beds, drip stands, bed pans, blood pressure machines, and thermometers from Project C.U.R.E. The district was engaged to integrate the supervision of equipment for MNCH and support future replacements and repairs.

### Health Information Systems

At 23 high-volume health facilities (>100 deliveries per month) in the 7 SMGL-supported districts, data were entered into the Pregnancy Outcomes Monitoring System (POMS), a database in Microsoft Access that includes every mother who delivered in a health facility and the outcome of every pregnancy. The Rapid Assessment of Pregnancy-Associated Institutional Death (RAPID) paper-based tool was used to ascertain all maternal deaths that occurred at high-volume facilities on a quarterly basis. This assessment was conducted by an external team of project staff in conjunction with the MNCH improvement team. Results of the RAPID tool were used to confirm the maternal deaths captured in the POMS database. These data were vital to guide program implementation and timely reporting. Midwives and records officers were trained in the use of POMS and the RAPID tool for improvement.

Data improvement committees comprised facility and department in-charges, and records officers were established to meet monthly to review these data and discuss progress and data-quality issues. These officers also disseminated best practices between facilities and other stakeholders.

Quarterly aggregated data from participating public and private not-for-profit facilities in a district were shared and feedback provided to individual facilities during quarterly data review meetings (Box 2). These data were validated with data from the District Health Information System 2 (DHIS2) and the national health information reporting system. The project also maintained a Microsoft Excel database with monthly facility process and outcome indicators from which time-series charts were generated to track progress for each site and district.

BOX 2 **Selected Saving Mothers, Giving Life Process and Output Indicators Monitored**

Some of the indicators that were tracked:

- Active management of third stage of labor
- Postpartum hemorrhage rate
- Correct partograph use to monitor labor
- Successful newborn resuscitation
- Essential newborn care package

The SMGL program strengthened reporting and data quality across all 118 supported facilities in the 7 SMGL-supported districts, with the 95 low-volume facilities providing MNCH program data through the national DHIS2 system. By December 2016, at the end of the SMGL program in Northern Uganda, the DHIS2 MNCH data were comparable with data from the POMS system in the 23 high-volume facilities. In the 9 unbranded SMGL-supported districts, the national DHIS2 system was strengthened through onsite mentorships and training in health management information system tools to collect quality data for reporting. Program performance in the unbranded SMGL-supported districts was assessed using DHIS2 data.

The SMGL program strengthened reporting and data quality across all 118 supported facilities in the 7 SMGL-supported districts.

SMGL also established and/or revitalized MPDR committees at 67 HC IIIs, HC IVs, and hospitals in the Phase 2 facilities. These committees were trained during onsite coaching and mentorship visits and supplied with MPDR forms. The MPDR committees in each facility met monthly and were supported in submitting their MPDR reports to the MOH through their respective district health offices. To further support facility team work on the MPDR recommendations, district MPDR committees were established in all 7 SMGL-supported districts. These committees met quarterly to review recommendations made at the facility level and develop district-based maternal and perinatal mortality reduction action plans. The committees were incentivized through promoting feedback from the district and MOH on all notified deaths as well as participating in stakeholder meetings. Maternal and perinatal death surveillance and response (MPDSR) is expected to be sustained in the future through the MPDSR guidelines, which regulate all actors at community, health facility, district, national, and other sector levels. The MPDSR committees oversee implementation of these guidelines at the facility, district, and national levels.

The SMGL established and/or revitalized MPDR committees at 67 HC IIIs, HC IVs, and hospitals in the Phase 2 facilities.

### Service Delivery

In line with the MOH national QI framework and strategic plan, QI teams were established in 118 health facilities to review MNH processes, identify gaps, implement solutions, and monitor performance. In the 20 high-volume facilities, hospitals and HC IVs, QI teams were trained to conduct MPDRs, provided with MPDR tools, and supported to address emerging gaps (Box 3).

BOX 3 **Community- and Facility-Level Interventions to Address the 3 Delays**

Community-Level Quality Improvement Interventions to Address the First and Second Delays

1. Ensure that women with pregnancy signs in the community attend their first antenatal care visit in the first trimester
2. Identify pregnant women with complications for management at the health facility
3. Ensure that every pregnant woman has a birth plan and saves for emergency birth expenses and delivery at the health facility
4. Encourage postnatal follow-up visits to check for mother and newborn wellness (days 2–3 and 4–7)

Facility-Level Quality Improvement Interventions to Address the Third Delay

1. Routine screening for complications during antenatal and labor and delivery care
2. Active monitoring of the labor process using a partograph
3. Active management of the third stage of labor
4. Helping Babies Breathe and essential newborn care packages provided
5. Preterm delivery management through antenatal corticosteroid use and kangaroo mother care
6. Establishment of and support to district and facility maternal and perinatal death review committees

CEmONC facilities were supported to perform cesarean deliveries. In Pajule HC IV (Pader District), Lalogi HC IV (Gulu District), and Ogur HC IV (Lira District), surgical theater functionality gaps were jointly identified with district leadership, and district resources were mobilized to address them (Box 4). The project facilitated training of 14 staff members in theater operating procedures at regional referral hospitals of Gulu and Lira. Laboratory teams from 2 HC IVs (Ogur and Amach) received support for training in blood transfusion services at the Gulu regional blood bank to obtain accreditation. Lalogi and Pajule HC IVs already had the capacity to perform blood transfusions.

BOX 4 **District-Led Initiatives to Make Functional Operating Theaters**

- In June 2015, the project team and the Gulu district health office (DHO) and the Gulu regional referral hospital jointly visited Lalogi and Awach health centers (HC IVs) to identify the critical issues affecting theater functionality
- A report was prepared by the Gulu DHO and presented to the district council
- The district council resolved to allocate funds in the subsequent financial year to conduct renovations of the 2 theaters
- In February 2016, renovation work of both theaters commenced at both HC IVs and was completed within 6 months
- The SMGL project supported the training of facility teams in theater operating procedures, surgical skills, and blood transfusions

Practices relating to improving care during labor and delivery, particularly the use of partograph and active management of third stage of labor, and newborn care, specifically improving newborn resuscitation and provision of the essential newborn care package, were adopted. For instance, special newborn care corners were established in 5 of 8 HC IVs, and the technical capacity for newborn intensive care was enhanced at 2 regional referral and 3 general hospitals.

### Community-Level Activities and Networks

Community-level activities included demand creation for quality MNH services, implementation of change packages, data collection and reporting, and strengthening of community networks. Community-level activities were led by village health teams (VHTs), the lowest level in the National Health Service delivery system. VHTs were responsible for mobilizing and promoting community participation through activities described in the following section.

### Community Activities to Address Delays 1 and 2

**FIGURE 5:**
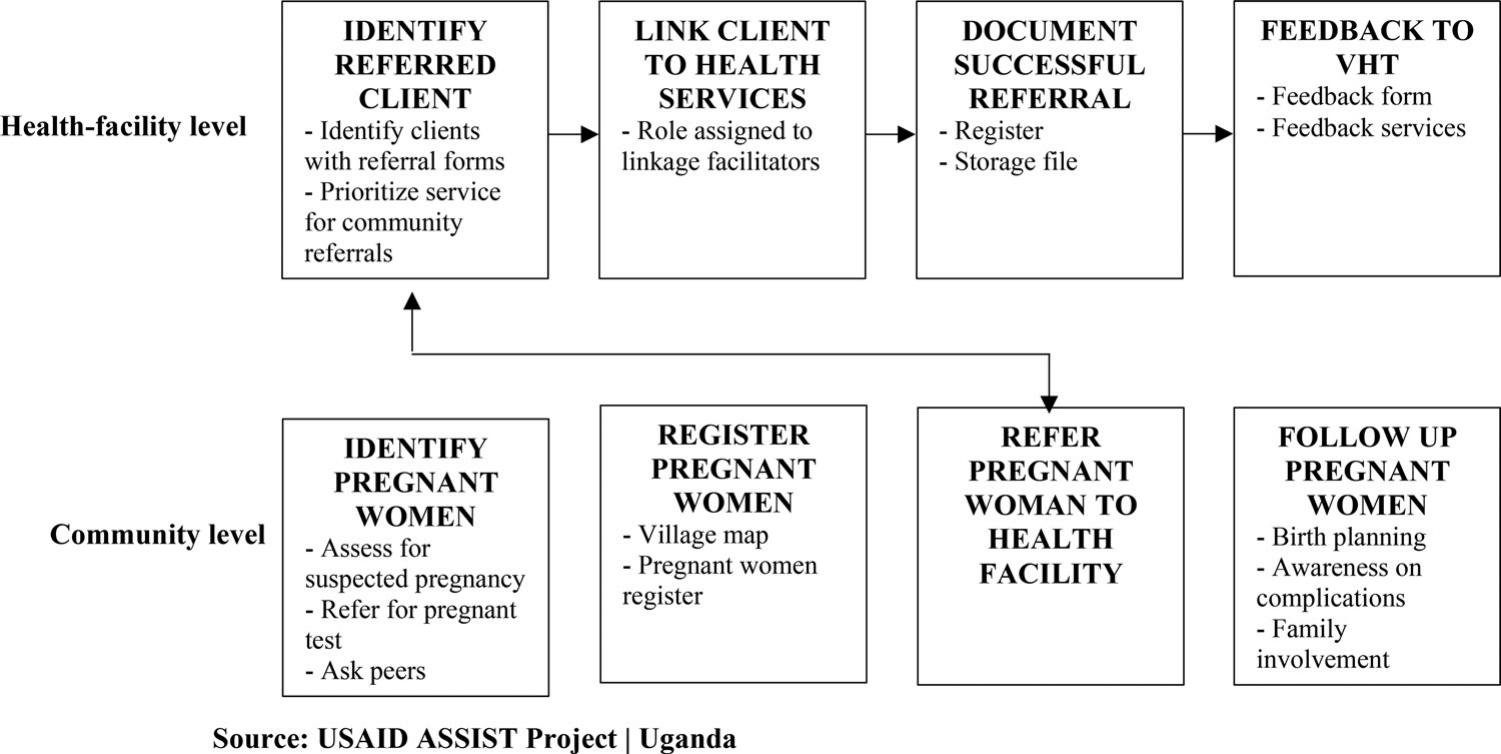
Health Facility and Community Care Pathway Developed Abbreviations: PW, pregnant woman; VHT, village health team.

The SMGL initiative in the learning districts developed materials and tools to support behavior change communication, such as radio talk shows, job aids, and posters. VHTs worked with resource persons and utilized gatherings at worship places, ceremonies, and other events and places to disseminate information about ANC and facility deliveries. Some community dialogue sessions were targeted to communities with low coverage of facility deliveries. Interpersonal communication was used during the home-to-home approach to reach out to individual postpartum and pregnant mothers. This approach was used to address individual needs and barriers to health seeking. A community-facility care pathway was designed by USAID Applying Science to Strengthen and Improve Systems (ASSIST) Project to standardize care for pregnant women and newborns (Figure 5). This pathway linked VHT activities to the facility and vice versa—with the facilities supervising VHT work—and formed the basis for community improvement team members, community processes, gaps analysis, monitoring, and coaching.

Through the SMGL intervention, VHTs mapped women of reproductive age (15 to 49 years) within their catchment area as well as community resources such as markets, religious houses, and traditional birth attendants that could all aid in increasing first ANC visits during the first trimester and health facility deliveries. Knowing where women lived and who they listened to facilitated identification of pregnancies through home-to-home visits and community engagements.

Knowing where women lived and who they listened to facilitated identification of pregnancies through home-to-home visits and community engagements.

The program supported the formation and functionality of QI teams at the village, parish, subcounty, and district levels; supervision followed the same structures. The teams engaged health facility service providers and community health workers—as VHTs—in implementing interventions to increase first ANC visits and facility deliveries. Community-level trainings were conducted that empowered VHTs with knowledge, skills, and tools for identifying, referring, and supporting women with suspected pregnancies. The VHTs then conducted home-to-home visits, registering women with suspected pregnancies and referring them to health facilities for pregnancy testing and first trimester ANC visits.

To allay myths and misconceptions surrounding early disclosure of pregnancy, which presents a barrier in first ANC uptake, the program supported community-level dialogue meetings and health education talks that engaged local leaders, pregnant women, male partners and mothers-in-law, health facility service providers, VHTs, traditional birth attendants, and district health office personnel.

Using a job aid, the project trained VHTs on the common danger signs in pregnancy and provided timely health facility referrals to pregnant women presenting with these signs. Every pregnant woman was supported in having a birth plan and saving for emergency birth expenses and delivery at a health facility. During the SMGL-supported community dialogue meetings with pregnant women, their partners, and local leaders, midwives and VHTs provided women with a list of essential items for labor and delivery. They also provided information on the cost of emergency transport and linked the women to existing village loans and savings associations for birth-related savings.

On average, pregnant women were saving US$20 to $30 for birth expenses. During each ANC visit, midwives checked to see how the women were progressing in acquiring birth items; a community follow-up was later conducted by the VHTs. To enhance emergency transportation of pregnant mothers in labor, contact information for reliable Boda-Boda riders (a motorbike taxi) and district ambulance drivers was shared during SMGL-supported community meetings.

Across the 7 SMGL-supported districts, the program engaged traditional birth attendants together with local leaders in meetings where midwives illustrated the risks of home deliveries for the mother and newborn. Traditional birth attendants were mapped according to villages and each was linked to a VHT in their catchment for follow-up. In front of their leaders, they committed to stop delivering women and, instead, committed to referring suspected pregnant women to the health facility for early ANC and escorting them to hospital for delivery.

## METHODS

### Wave-Sequence Approach

The wave-sequence approach focuses on spreading improved care delivery to other parts of a system. The term “wave” indicates that this method of spread occurs sequentially to reach increasingly larger sections of a health care system. Wave-sequence spread is used when it is not possible to cover the whole system all at once.^6^ Initial work in Wave I focused on improving quality of care and developing a team of regional mentors and coaches to support spread in Waves II and III. The community-level work began in Wave I, with interventions targeting 144 communities in the catchment areas of 16 of 20 high-volume facilities. Wave II involved deploying regional mentors and other learning platforms. Effective changes were spread from the 20 initial sites to 60 spread facilities, and later to 38 Wave III facilities. Wave II focused on HC IIIs and Wave III on HC IIs. In a similar way, community interventions were also spread to 370 communities attached to 32 out of 98 spread facilities in the first wave, with the intention of identifying best practices and scaling up for the remaining communities in Waves II and III.

### Program Evaluation

A program evaluation was carried out to obtain insights on how QI influenced improvements in care seeking, service provision, and reduction in maternal mortality in the communities and health facilities within the 7 SMGL Phase 2 districts. The evaluation was a facility- and community-based cross-sectional design and used a mixed-methods approach to enrich information on how QI activities influenced improvements. Both purposive and random selection procedures were used to obtain a reasonable representation of the different study groups and their related contextual conditions. In August 2016, cross-sectional data were collected through interviews with a random sample of MNH care providers and postpartum and pregnant mothers from select Phase 2 health facilities and communities, using a structured interview questionnaire.

A program evaluation obtained insights on how QI influenced improvements in care seeking, service provision, and reduction in maternal mortality in the communities and health facilities within the 7 SMGL Phase 2 districts.

The sample of facilities was determined based on the level of health facility, monthly volume of deliveries, and anticipated proportion of facilities with attributes of interest, such as those that offered delivery services and were in the intervention sites. Other factors included the alpha error, confidence level, and margin of error. Thus, given a confidence level of 95% (1.96 confidence interval), margin of error (15%), *p* as the anticipated proportion of facilities with the attribute of interest (.5) *q* = 1 − *p*, and design effect, the evaluation covered 32 health facilities. The sample was adjusted to each facility type to accommodate both low- and high-volume facilities.

The sample for providers was estimated by considering the number and level of selected facilities, provider selection criteria, and the general human resource capacity at the facilities. Health workers who provide MNH services in each of the selected facility were interviewed. A total of 125 MNH care providers were purposively selected from 25 intervention facilities from the 7 SMGL Phase 2 districts.

A sample of 103 postpartum and pregnant mothers were purposively selected from 12 intervention communities in 7 SMGL Phase 2 districts. These mothers had more than 1 birth, with their last birth occurring within the intervention period (2015 to 2016). In computing the sample size for mothers, we considered the potential to use the sample to explore differences within the same group and the potential to assess different outcomes. The sample size was determined using the formula for the calculation of sample size in populations and thus considered a standard normal deviation of 95% confidence interval of 1.96 (*z*); proportion of women who attend ANC (*p*); the complementary probability of *P* (1 − *p*), that is, the percentage of women not attending antenatal visits (*q*); and alpha error of 5%. It is estimated that about 90% to 93% of pregnant women in Uganda had at least 1 ANC visit and 48% had 4 visits. With an expected response rate of 96%, an adjustment of the sample size estimate to cover for nonresponse was made by dividing the sample size calculated with a factor *f*, that is, *n*/*f*, where *f* is the estimated response rate. This process yielded a desired sample size of 104 mothers.

The SMGL facility component was assessed on the following domains: gains/value added to the quality of the work of health care providers, sustainability of SMGL activities, and the effect of SMGL activities on the quality of services and maternal deaths over the intervention period. Among beneficiaries (postpartum and pregnant mothers), emphasis was on understanding their experiences in terms of quality of services and satisfaction with provider services at the intervention facilities.

Quantitative data were analyzed using the Statistical Package for the Social Sciences for Windows version 16.0 (SPSS Inc., Chicago, IL, USA). Univariate analysis was conducted to describe the experiences of mothers and health care providers in SMGL-supported communities and health facilities, respectively. Descriptive statistics were obtained on quantifiable variables under assessment. Qualitative data with health care providers and mothers were transcribed verbatim into full text and coded using a thematic analysis to illuminate the context of interventions as a whole. The quotations included in the text best represent the range of ideas voiced around key themes and were edited without altering the meaning or violating anonymity.

## RESULTS

### Trends in Maternal Death Ratios and Newborn Death Rates

Trends in maternal death ratios and newborn death rates in the SMGL-supported districts of Northern Uganda were determined based on data from POMS and RAPID for the 23 high-volume facilities of the 7 SMGL-supported districts, and the results were triangulated with data from the national DHIS2 system. These data were also compared with DHIS2 data for a random sample of other districts in the region without SMGL interventions to estimate the effect of the interventions on maternal and newborn mortality. In addition, DHIS 2 data from the preintervention period (2014) in SMGL-supported Phase 2 districts were used as a baseline to estimate the effect of the intervention.

For the intervention districts, data for each indicator were computed to estimate the effect of the intervention and then compared with results in the DHIS2 for validation. DHIS2 results also documented maternal and newborn mortality in both SMGL Phase 2 and unbranded SMGL-supported districts to determine whether a reduction occurred in newborn and maternal deaths in SMGL-supported districts compared to the unbranded SMGL-supported districts in selected comparator facilities, using data captured in the national health management information system. Additional analysis was conducted to quantify the coverage of different services given to the mothers and their newborns at the intervention sites. Thus, the indicators used were not district level but reflect outcome data in these selected facilities.

Data in the national DHIS2 system, summarized in Table 2, show that the facility MMR in the 7 SMGL-supported districts in Northern Uganda of Phase 2 decreased by 21%, from 138 to 109 maternal deaths per 100,000 live births, between December 2014 and December 2016. Nationally, the institutional MMR increased between 2014 and 2016 from 124 to 181 maternal deaths per 100,000 live births. With the spread of SMGL best practices in January 2016, institutional maternal mortality in the unbranded SMGL-supported districts in Northern Uganda was also reduced by nearly 22%, from 106 to 83 maternal deaths per 100,000 live births between 2015 and 2016.

**TABLE 2.** Summary of Key Maternal and Newborn Outcomes in National, SMGL, and Unbranded SMGL districts, 2014–2016^a^

	National			Unbranded SMGL			SMGL		
	2014	2015	2016	2014	2015	2016	2014	2015	2016
Maternal death									
Deliveries	862,538	1,122,838	998,162	42,218	47,215	45,721	45,631	49,174	50,521
Maternal deaths	1,071	1,543	1,807	25	50	38	63	60	55
Institutional MMR/100,000	124	137	181	59	106	83	138	122	109
Perinatal death									
Total births	845,877	936,790	980,574	41,914	46,591	45,629	45,697	48,814	49,855
Total perinatal deaths	27,464	33,589	30,248	1,064	1,476	1,059	1,529	1,628	1267
PNMR/1,000 total births	32.5	35.9	30.8	25.4	31.7	23.2	33.5	33.4	25.4
Predischarge newborn deaths									
Total live births	825,303	914,387	958,398	41,137	45,740	45,011	44,482	47,455	48,831
Total early newborn deaths	6890	11,186	8,072	287	625	441	314	269	243
ENMR/1,000 live births	8.3	12.2	8.4	7.0	13.7	9.8	7.1	5.7	5.0
Fresh stillbirth									
Total births	845,877	936,790	980,574	41,914	46,591	45,629	45,697	48,814	49,855
Total fresh stillbirths	12,213	12,531	11,156	496	507	288	720	744	419
FSBR/1,000 total births	14.4	13.4	11.4	11.8	10.9	6.3	15.8	15.2	8.4

Abbreviations: ENMR, early newborn mortality rate; FSBR, fresh stillbirth rate; MMR, maternal mortality ratio; PNMR, perinatal mortality rate; SMGL, Saving Mothers, Giving Life.

a Rates are facility based.

In the 7 Phase 2 SMGL-supported districts in Northern Uganda, MMR decreased by 21%, from 138 to 109 maternal deaths per 100,000 live births between 2014 and 2016.

Between 2014 and 2016, facility perinatal mortality rate was reduced by 25%, from 31.6 to 23.6 perinatal deaths per 1,000 live births, in the 7 SMGL-supported districts according to the DHIS2 (Table 2). With the spread of SMGL best practices to the 9 unbranded SMGL-supported districts from January 2016, the perinatal mortality rate fell by 25%, from 31.7 perinatal deaths per 1,000 live births in 2015 to 23.2 perinatal deaths per 1,000 live births by December 2016. Nationally, perinatal mortality dropped between 2014 and 2016 (Table 2).

The predischarge newborn mortality rate also declined faster in the 7 SMGL-supported districts in Northern Uganda than the national average (Table 2). Predischarge newborn deaths were reduced by 30%, from 7.1 to 5.0 deaths per 1,000 live births between 2014 and 2016 in the 7 SMGL-supported districts of Northern Uganda. With the spread of SMGL best practices, the early newborn mortality rate was reduced by 28%, from 13.7 deaths per 1,000 live births in 2015 to 9.8 deaths per 1,000 live births in 2016. Nationally, predischarge neonatal deaths remained unchanged from 2014 to 2016, despite an increase in 2015 (Table 2).

The fresh stillbirth rate was reduced by 47%, from 15.8 to 8.4 stillbirths per 1,000 births between 2014 and 2016 in the 7 SMGL-supported districts (Table 2). With the spread of SMGL best practices, the fresh stillbirth rate was reduced by 43%, from 10.9 to 6.2 stillbirths per 1,000 births between 2015 and 2016 in the 9 unbranded SMGL-supported districts. Nationally, fresh stillbirths also declined from 2014 to 2016, but to a smaller degree (21%) (Table 2). It is important to note that some mortality rates in unbranded SMGL-supported districts were lower in 2014 than in 2015 or even in 2016. This is likely because these districts were newly carved out from the old districts with a small number of facilities and population.

### Improvements in Service Delivery

In Phase 2 facilities, over 90% of women were screened for hypertension and about 70% of women were screened for syphilis during ANC. All women received a uterotonic drug to prevent postpartum hemorrhage during delivery and about 90% of women were monitored using a partograph during labor. About 92% of babies not breathing spontaneously at birth were resuscitated successfully. Over 95% of newborns were discharged having received all elements of the essential newborn care package: cord care, eye care, skin-to-skin contact, initiation of breastfeeding within 1 hour of birth, thermal care, administration of injectable vitamin K, immunization with bacilli Calmette-Guerin and polio vaccines, and screening for infections.

The institutional delivery rate in the 118 facilities in the 7 Phase 2 SMGL-supported districts increased by 5.2% between 2014 and 2016. Access to cesarean delivery also increased as 3 more HC IVs began offering this service, giving a total of 8 facilities providing this intervention. Overall, the facility-based cesarean delivery rate increased from 3.9% to 4.2% of deliveries. The number of mothers receiving antiretroviral therapy (option B+) for the prevention of mother-to-child transmission of HIV increased by 39.9% in these facilities, and the rate of low birthweight was reduced by 31.8%.

### The Effect of QI on Care Seeking and Service Delivery

**FIGURE 6:**
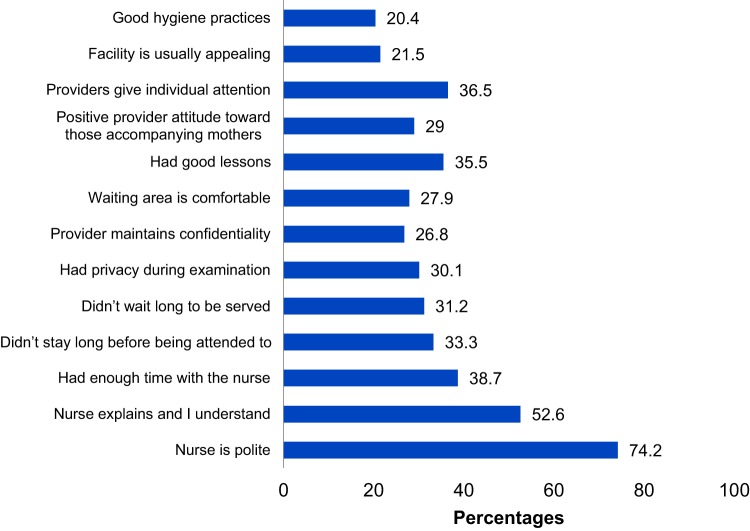
Postpartum and Pregnant Mothers' Experiences With Antenatal Care Services (n=103)

The 103 postpartum and pregnant mothers were asked about their experiences with the services provided at a health facility during ANC to assess service satisfaction from the QI interventions. The provider was central to their assessment of quality of services at the facility. Politeness of nurses (n=76, 74.2%), clear explanations provided by nurses during health talks (n=54, 52.6%), and time spent with the nurses (n=40, 38.7%) stood out as positive experiences (Figure 6).

When asked about the health workers' attitudes toward mothers at ANC, mothers expressed that providers were receptive and described them as welcoming, good, friendly, and caring. These comments resonate with the results indicating an increase in use of ANC and health facility delivery, described above, and are validated by the following comments:

*They were positive [health care providers]. I still had blood coming out from me. They prescribed drugs and told me to return for follow-up.* (Mother from Apac)

*They treat mothers with love and respect. They are also happy when the baby is clean and healthy.* (Mother from Apac)

*They [health care providers] are always understanding and educate us [mothers] on what to do and how to eat in order to regain the lost blood.* (Mother from Dokolo)

*They have a good attitude, but they treat you according to how you are caring about your child, for example, washing the baby's clothes.* (Mother from Nwoya)

When asked about the health workers' attitudes toward mothers at ANC, mothers expressed that providers were receptive and described them as welcoming, good, friendly, and caring.

Postpartum and pregnant mothers were asked about their impression of the services at their last ANC visit and delivery, compared to their previous birth. They largely pointed to a positive change in the quality of services and expressed that health workers were more polite during the most recent pregnancy:

*In the past, health workers were rude to me [mother], but these ones [health care providers] are humble and polite.* (Mother from Gulu)

*[T]he current ones are more caring than the previous ones. In fact there is an improvement in the quality of services. The services are better compared to the previous ones.* (Mother from Apac)

*It [health care services] was much better this time because health workers seem to work faster on the pregnant women.* (Mother from Nwoya)

### Postpartum and Pregnant Mothers' Experience of VHT Work

**FIGURE 7:**
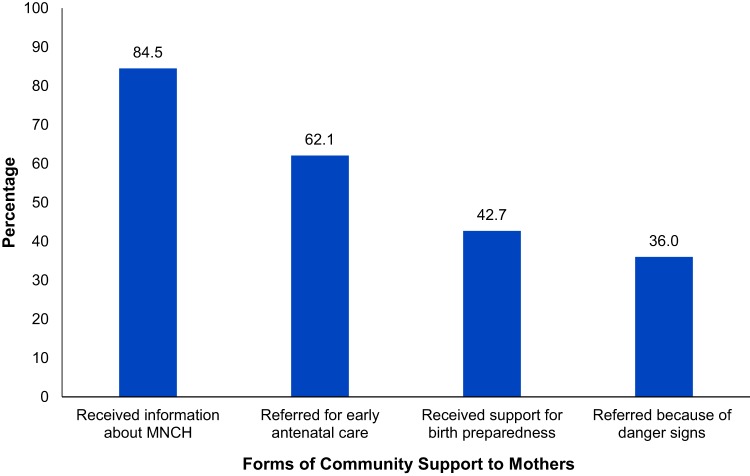
Forms of Community Support to Postpartum and Pregnant Mothers (n=103) Abbreviation: MNCH, maternal newborn, and child health.

In Figure 7, mothers were asked about the form of support received from the community health workers (VHTs). More than 80% had received information about maternal and newborn care from VHTs and 62% (n=64) had been referred for early ANC by VHTs. Only 36% (n=37) were referred by VHTs because of pregnancy danger signs. More than two-fifths (43%) of VHTs were reported as supporting postpartum and pregnant mothers in preparing for birth.

### Effect of QI Strategies on MNH Services Over the Intervention Period

Following implementation of QI strategies, health care providers in the Phase 2 districts were asked to look back to the period before SMGL activities at their respective facilities and compare MNH services before and after SMGL. All (n=125, 100%) of the providers stated that the quality of service delivery improved and maternal and newborn deaths were reduced at their facility. All of the providers also reported that facility deliveries improved in the previous year, which they mainly attributed to the home-to-home activities of the VHTs and use of ANC charts during their health education talks with women and other villagers.

All providers stated that the quality of service delivery improved and maternal and newborn deaths were reduced at their facility.

The use of partographs to monitor women during labor improved following SMGL interventions with more than 50% (n=63) of providers reporting attaining skills in use of partograph through the mentorship approach. This is illustrated by a comment from a health care provider in Nwoya:

*I learnt how to carry out syphilis test and to use a partograph for monitoring mothers during labor.* (Health care provider from Nwoya)

Three-quarters of the health care providers (n=94, 75%) cited a reduction in the number of mothers referred to a higher-level facility, as illustrated by the following comment:

*There are now a few mothers being referred for higher level care from this facility*. (Health care provider from Gulu)

### Effect of QI Strategies on Provider Skills

All of the health care providers (n=125, 100%) reported that SMGL improved the way they work, citing their skills and standard of work as being improved due to SMGL coaching/mentorship:

*There is increased interest in work because with the skills and knowledge gained, work can be easier.* (Health care provider from Apac)

All health care providers reported that SMGL improved the way they work, citing their skills and standard of work as being improved due to SMGL coaching/mentorship.

Health workers also indicated that they gained skills in the uptake of evidence-based practices, such as use of partograph, newborn resuscitation, documentation, and data use to inform decisions. This gain is expressed in the following comment:

*I am able to monitor my work through documentation journal.* (Health care provider from Gulu)

However, more than half (n=80, 64%) of providers expressed concerns about an increased workload resulting from implementation of QI activities. Directly, mentorship activities took time that would otherwise be spent with mothers. Indirectly, more mothers came to facilities following QI activities at the community level, yet staffing levels remained constant.

### Sustainability of QI Activities at Facility and Community Levels

The majority (n=112, 90%) of providers thought that all SMGL activities could be sustained since the QI strategies are in the required guidelines for MNH. The following items were repeatedly singled out as possible areas for continuity: use of partograph, referral of mothers from communities, continuous medical education in place of SMGL onsite training, documentation, use of emergency tray, kangaroo method of care, and use of tests, such as HIV testing, urinalysis, and blood pressure checks. This finding is expressed in the following quotations:

*Active management of the third stage of labor, use of partograph, essential newborn care and all activities shall continue at this facility.* (Health care provider from Dokolo)

*All activities can be sustained as long as you do it properly.* (Health care provider from Gulu)

*All activities can be sustained*. (Health care provider from Nwoya)

*The quality improvement teams will continue.* (Health care provider from Apac)

All VHTs (n=36, 100%) expressed continuity of 1 or more QI activities within their mandate since all aspects of their work are voluntary. Such activities included house-to-house visits by VHTs, health education, and referral. Asked about how these activities can be sustained, providers suggested taking advantage of the already established relationships with the clergy to educate the population and using their location to reach women within their own communities, as illustrated by the following statements:

*Following up these mothers and referral will remain since we as VHTs, we stay with these mothers in the community*. (VHT from Lira)

*We will continue sensitizing the women about facility delivery and ANC*. (VHT from Gulu)

*We shall remain working even when the project ends*. (VHT in Pader)

### Challenges Experienced in Implementing QI Strategy

**FIGURE 8:**
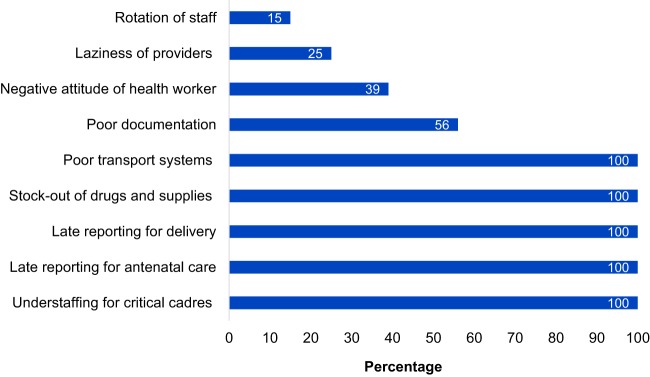
Common Challenges Faced in Implementation of Quality Improvement Strategies (n=125)

Health care providers expressed the most common challenges were associated with workload due to understaffed critical cadres, rotation of staff, poor documentation, late reporting of mothers for delivery, long distances to the facility, inadequate resources, occasional stock-out of partographs, and inconsistent supply of drugs, mama kits, and mosquito nets (Figure 8).

**FIGURE 9:**
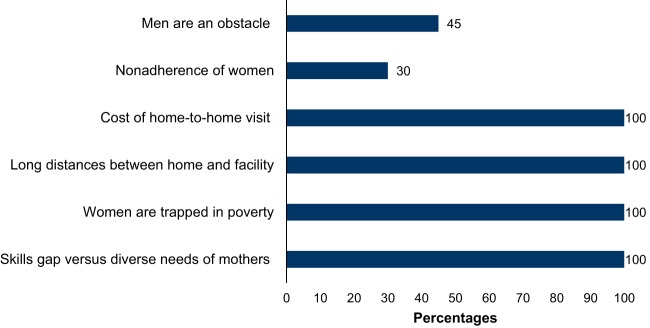
Common Challenges Faced by Village Health Teams in Percentages (n=125)

At the community level, challenges were documented, such as limited VHT skills and knowledge of MNH, while the health needs of the women are diverse; their work is hindered by inadequate resources for the women, especially transport to the health facility; long distance from home to the facility makes it hard for referred mothers to keep their ANC appointments; nonadherence of mothers to VHT guidance; and poverty in the community (Figure 9).

## DISCUSSION

QI approaches are increasingly being used in low- and middle-income countries to strengthen health systems and to improve service delivery and health outcomes. Much of the literature on QI approaches in these settings focuses on documentation of implementation and process evaluation.^8^ A few studies, such as one in Ecuador,^9^ have described scale-up processes for QI interventions. This paper describes an intervention that used a wave-path approach.

QI approaches are increasingly being used in low- and middle-income countries to strengthen health systems and to improve service delivery and health outcomes.

In Ghana, a quasi-experimental, pre- and post-intervention analysis of the QI intervention analyzed system deficiencies and 97 improvement activities that were implemented between January 2007 and December 2011. Data were collected on outcomes and implementation rates of improvement activities. Maternal mortality decreased by 22% between 2007 and 2011, from 496 to 385 maternal deaths per 100,000 live births, despite a 50% increase in deliveries and 5- and 3-fold increases in the proportion of pregnancies complicated by obstetric hemorrhage and hypertensive disorders of pregnancy, respectively.^10^ This multitiered QI strategy in Ghana showed that within 7 months of introduction of a QI program to triage sick mothers and to clean and organize the neonatal intensive care unit to reduce errors, there was a 4-fold reduction in the percentage of mothers needing emergency cesarean surgery with unacceptable waiting times, over 93% accuracy in identification of the sickest mothers, and a 37% increase in hand hygiene compliance.^10^

In our intervention using the wave approach to scale up QI in Phase 2 districts, a decline in maternal and perinatal mortality within 2 years was associated with the project's activities to strengthen the health system. Similar results were not observed in the unbranded SMGL-supported districts in Northern Uganda until 2016, when best practices from the Phase 2 SMGL intervention districts were spread to 9 other districts in Northern Uganda.

Between December 2014 and December 2016, data from the Uganda DHIS2 systems showed that institutional MMR decreased by 20%, from 138 to 109 maternal deaths per 100,000 live births in 2016 in the Phase 2 facilities. In the 9 unbranded SMGL-supported districts of Northern Uganda, institutional maternal mortality was reduced by 21% to 75 maternal deaths per 100,000 live births with the spread of SMGL best practices starting in January 2016.

Between December 2014 and December 2016, data from the Uganda DHIS2 systems showed that institutional MMR decreased by 21%, from 138 to 109 maternal deaths per 100,000 live births in Phase 2 facilities.

Newborn deaths have also been reported to decline with QI techniques. Endline analysis to assess the standard of neonatal care found a reduction in mortality rate among newborns admitted to Central Beira Hospital's Neonatal Intensive Care Unit after the first year of the Doctors with Africa CUAMM intervention in Mozambique. Most of this reduction was attributed to the decrease in deaths from asphyxia, sepsis, and prematurity.^11^

In the Uganda Phase 2 districts, we found a decline in the perinatal mortality rate (stillbirth plus pre-discharge/early newborn mortality) between 2014 and 2016. A decrease of 25% was observed in the 7 SMGL-supported districts, from 31.6 to 23.6 deaths per 1,000 births between 2014 and 2016. The reduction in stillbirths implied improvement in the quality of labor and delivery care. In the 9 unbranded SMGL-supported districts of Northern Uganda, the perinatal mortality rate declined by 25% within 1 year with the spread of SMGL best practices starting in January 2016. System-strengthening activities addressing the main gaps in service delivery through the work of QI teams, supported by district management and QI coaching from the USAID ASSIST Project, were the most important factors explaining the observed results. The ASSIST Project provided QI technical assistance to Uganda's MOH and implementing partners with the overall goal of providing quality health services and building a system through which these services can be delivered in a sustainable way.

It is widely agreed that communities should take an active part in improving their own health outcomes.^12^ Although strategies vary considerably, community-based interventions may encompass encouraging healthier practices and care seeking among communities and families, recruiting and training local community members to work alongside trained health care professionals, and community member involvement in service provision, including diagnosis, treatment, and referral. A range of approaches exist within these broad categories, including community health workers, traditional birth attendants, health campaigns, school-based health promotion, home-based care, and even community franchise-operated clinics.^13^ In the Northern Uganda districts, as the health care service delivery systems improved, the uptake of facility deliveries also increased, supported by the community component of the intervention. VHTs educated mothers on the importance of timely ANC and facility deliveries, which resulted in increases in early ANC attendance and births in facilities.

It is widely agreed that communities should take an active part in improving their own health outcomes.

The use of data and the improvements in the health management information system led to identification of priorities for targeted QI efforts that led to improvements in the quality of care. The established monitoring and evaluation activities of the SMGL initiative and functionalized data improvement committees at facility and district levels conducted meetings on a monthly and quarterly basis, respectively. Death reviews informed facility teams on the causes of death that led to further QI activities. For example, Helping Babies Breathe skills laboratories were established and supported in selected high-volume facilities where newborn deaths were high due to birth asphyxia, improving staff skills in newborn resuscitation. Collectively, these interventions were associated with reductions in maternal and perinatal deaths within the relatively short period of the intervention.

In settings with limited registration of births and deaths and incipient health information systems, monitoring of maternal mortality is largely done through model-based estimates.^3^ POMS, RAPID, and national DHIS2 systems were direct sources for estimating the effect of the intervention by contrasting maternal deaths in the intervention and unbranded SMGL-supported facilities prior to and after the spread of SMGL best practices. These data sources were used to track changes in service delivery and maternal deaths resulting from the intervention.

The interventions were rolled out by QI teams operating at different levels, including district, health facility, and community. Our results suggest that to kick-start improvement activities and achieve impact in a short period of time, implementers need to identify critical elements of maternal and newborn care package; define standard care pathways in the community and at the health facility; communicate with and engage key actors at each step in the process; form improvement teams comprising the different actors in each care step; use champions at district, facility, and community levels to scale up and support other sites; identify and review data regularly for decision making; and use a systematic scale-up strategy to spread better practices throughout a large geographic area.

## CONCLUSION

A participatory health system strengthening approach applied in the SMGL initiative in the Northern region of Uganda had a positive effect on reducing institutional maternal and newborn mortality. Working at all levels of the health system not only strengthens the system but also fosters better performance through supportive leadership, engaged improvement teams, well-supplied facilities, trained health workers, and social support networks. Linking community activities with health facilities is a key strategy for reaching target populations and improving care seeking. The participatory nature and skill-building potential of improvement activities was important for overcoming barriers to quality of care associated with limited skills.

## Notes

**Peer Reviewed**

**Funding:** This study and the intervention described were made possible by the support of the American people through PEPFAR and USAID and were implemented by the USAID ASSIST Project, managed by University Research Co., LLC under Cooperative Agreement Number AID-OAA-A-12-00101.

## References

[1] World Health Organization (WHO), United Nations Children's Fund, United Nations Population Fund, World Bank Group, and the United Nations Population Division. Trends in Maternal Mortality: 1990 to 2015. Geneva: WHO; 2015 https://www.who.int/reproductivehealth/publications/monitoring/maternal-mortality-2015/en/ Accessed December 14, 2018.

[2] Uganda Bureau of Statistics (UBOS) and ICF. Uganda Demographic and Health Survey 2016. Kampala, Uganda, and Rockville, MD: UBOS and ICF; 2018 https://dhsprogram.com/pubs/pdf/FR333/FR333.pdf Accessed January 1, 2019.

[3] Say L Chou D Gemmill A Global causes of maternal death: a WHO systematic analysis. Lancet Glob Health. 2014;2(6):e323–e333. 10.1016/S2214-109X(14)70227-X 25103301

[4] Ministry of Health (MOH). Annual Health Sector Performance Report: Financial Year 2013/2014. Kampala, Uganda: MOH: 2014 http://www.nationalplanningcycles.org/sites/default/files/planning_cycle_repository/uganda/final_ahspr_2013_2014.pdf Accessed December 14, 2018.

[5] Serbanescu F Goldberg HI Danel I. Rapid reduction of maternal mortality in Uganda and Zambia through the saving mothers, giving life initiative: results of year 1 evaluation. BMC Pregnancy Childbirth. 2017;17:42. 10.1186/s12884-017-1222-y. 28103836 PMC5247819

[6] Massoud MR Mensah-Abrampah N. A promising approach to scale up health care improvements in low-and middle-income countries: the Wave-Sequence Spread Approach and the concept of the Slice of a System. F1000 Res. 2014;3:100. 10.12688/f1000research.3888.1. 25309727 PMC4184307

[7] World Health Organization (WHO). Everybody's Business: Strengthening Health Systems to Improve Health Outcome: WHO's Framework for Action. Geneva: WHO; 2007 http://www.who.int/healthsystems/strategy/everybodys_business.pdf Accessed December 14, 2018.

[8] Leatherman S Ferris TG Berwick D Omaswa F Crisp N. The role of quality improvement in strengthening health systems in developing countries. Int J Qual Health Care. 2010;22(4):237–243. 10.1093/intqhc/mzq028. 20543209

[9] Hermida J Salas B Sloan NL. Sustainable scale-up of active management of the third stage of labor for prevention of postpartum hemorrhage in Ecuador. Int J Gynaecol Obstet. 2012;117(3):278–282. 10.1016/j.ijgo.2012.01.017. 22483573

[10] Srofenyoh EK Kassebaum NJ Goodman DM Olufolabi AJ Owen MD. Measuring the impact of a quality improvement collaboration to decrease maternal mortality in a Ghanaian regional hospital. Int J Gynaecol Obstet. 2016;134(2):181–185. 10.1016/j.ijgo.2015.11.026. 27177512

[11] Cavicchiolo ME Lanzoni P Wingi MO. Reduced neonatal mortality in a regional hospital in Mozambique linked to a Quality Improvement intervention. BMC Pregnancy Childbirth. 2016;16:366. 10.1186/s12884-016-1170-y. 27876013 PMC5120470

[12] World Health Organization (WHO). Rio Political Declaration on Social Determinants of Health. Rio de Janeiro, Brazil: WHO; 2011 https://www.who.int/sdhconference/declaration/Rio_political_declaration.pdf?ua=1 Accessed January 1, 2019.

[13] Scott S Kendall L Gomez P. Effect of maternal death on child survival in rural West Africa: 25 years of prospective surveillance data in The Gambia. PLoS One. 2017;12(2):e0172286. 10.1371/journal.pone.0172286. 28225798 PMC5321282

